# Multi–omic analysis of signalling factors in inflammatory comorbidities

**DOI:** 10.1186/s12859-018-2413-x

**Published:** 2018-11-30

**Authors:** Hui Xiao, Krzysztof Bartoszek, Pietro Lio’

**Affiliations:** 1 Computer Laboratory, University of Cambridge, Cambridge, UK; 2 Department of Computer and Information Science, Linköping University, Linköping, Sweden

**Keywords:** Multi–omic approach, Gene families, Evolution, Inflammation

## Abstract

**Background:**

Inflammation is a core element of many different, systemic and chronic diseases that usually involve an important autoimmune component. The clinical phase of inflammatory diseases is often the culmination of a long series of pathologic events that started years before. The systemic characteristics and related mechanisms could be investigated through the multi–omic comparative analysis of many inflammatory diseases. Therefore, it is important to use molecular data to study the genesis of the diseases. Here we propose a new methodology to study the relationships between inflammatory diseases and signalling molecules whose dysregulation at molecular levels could lead to systemic pathological events observed in inflammatory diseases.

**Results:**

We first perform an exploratory analysis of gene expression data of a number of diseases that involve a strong inflammatory component. The comparison of gene expression between disease and healthy samples reveals the importance of members of gene families coding for signalling factors. Next, we focus on interested signalling gene families and a subset of inflammation related diseases with multi–omic features including both gene expression and DNA methylation. We introduce a phylogenetic–based multi–omic method to study the relationships between multi–omic features of inflammation related diseases by integrating gene expression, DNA methylation through sequence based phylogeny of the signalling gene families. The models of adaptations between gene expression and DNA methylation can be inferred from pre–estimated evolutionary relationship of a gene family. Members of the gene family whose expression or methylation levels significantly deviate from the model are considered as the potential disease associated genes.

**Conclusions:**

Applying the methodology to four gene families (the chemokine receptor family, the TNF receptor family, the TGF– *β* gene family, the IL–17 gene family) in nine inflammation related diseases, we identify disease associated genes which exhibit significant dysregulation in gene expression or DNA methylation in the inflammation related diseases, which provides clues for functional associations between the diseases.

**Electronic supplementary material:**

The online version of this article (10.1186/s12859-018-2413-x) contains supplementary material, which is available to authorized users.

## Background

Inflammation is the body’s attempt at removing harmful or irritating affects, which is part of the body’s immune response. The inflammatory response is essential for the recruitment and activation of lymphocytes in order to respond to an infection and the subsequent promotion of wound healing and repair. Strong intensity and long duration of unconstrained inflammatory response will cause the consequences of unregulated inflammation, which might result in many acute and chronic autoimmune diseases and comorbidities [1–4]. The inflammatory system is complex because of comorbidities, which involves depression, immune–inflammatory, oxidative stress, gut–brain pathways and so on [5]. For example, inflammation and altered gut microbiota (dysbiosis) could lead to colorectal cancer carcinogenesis [6, 7]. The severity of inflammatory diseases is strongly correlated with high levels of proinflammatory cytokine. Scientific evidence has shown that gut microbiota plays important roles in the genesis of several inflammatory diseases such as arthritis, systemic lupus erythematosus (SLE), pathogen induced colitis, Crohn’s disease, inflammatory bowel disease (IBD) [8–16]. Besides, inflammation has also been reported one of the enabling characteristics of cancer development such as colon cancer and breast cancer [17]. The genesis of cancers are considered to be related with the inflammatory responses to microbial or damaged-self stimuli. Both arms of immunity, innate and adaptive, play important roles during tumorgenesis. Growing attentions have been attracted in identifying early biomarkers for inflammatory diseases by exploring the associated molecular mechanisms [18], because the genesis of inflammatory diseases usually take a long preclinical period [19] and the identification of early disease markers could provide valuable clues for better clinical therapies. It is reported that a set of circulating proteins such as inflammatory cytokines and endocrine factors (e.g., TGF– *β*, TNF, and chemokines), forming a communicome, are involved in inter-cellular and organs communication, which are responsible for spreading inflammation in the body [20].

Recent advances in high–throughput genomics biotechnology such as microarrays and next generation sequencing have produced various omic data such as genome, epigenome, transcriptome, proteome and so on. The rapid growth of the amount of multi–omic data provides great opportunities to understand the mechanisms of complex biological systems such as human diseases from multiple molecular levels [21, 22]. For example, Zhang et al. [23] predicted the driver genes associated with different clinical outcome subtypes of ovarian cancer by integrating genome–wide gene expression, DNA methylation, microRNA expression and copy number alteration profiles. Cabezas-Wallscheid et al. [24] performed a comprehensive analysis of proteome, transcriptome and DNA methylome data to identify coordinated changes at the protein, RNA, and DNA levels during early differentiation steps of hematopoietic stem cells (HSCs). Cantini et al. [25] proposed a multilayer network community detection method to identify cancer related gene modules, which reveals cancer driver genes, through the integration of gene expression, protein interactome and transcription factor regulation network. Chaudhary et al. [26] introduced a neural network model to predict survival in liver cancer by integrating multi–omic data including gene expression, DNA copy number and miRNA expression data.

In order to explore the associations between signalling factors and inflammatory diseases as well as cancers, we propose a new methodology based on phylogenetic inference on multi–omic data to identify gene markers of diseases. Taking full advantage of the pre–estimated evolutionary relationship of a gene family with multi–omic information including gene expression and DNA methylation, it is capable of identification of genes exhibiting significant alterations in expression or methylation levels in diseases. A multi–omic approach is necessary as it integrates information from all sources. Phylogenetic information is important as some genetic behaviours may be due to evolutionary inertia. The phylogenetic correlations between gene expression and methylation help in identifying disease relationship due to perturbations of the same or closely related gene family members.

Applying the proposed method, we perform a comparative study of the signatures of signalling molecules in several inflammation related diseases, which consists of a two-step analysis: Firstly, we present a systematic study of genomewide molecular signatures, based on gene expression, for several inflammatory diseases as well as cancers. Most of the significant molecular signatures are related to members of a few important gene families. Then, we propose a phylogenetic–based multi–omic approach and apply it to four signalling related gene families selected from the first step to study the correlated or independent roles of the genes as disease markers by integrating the sequences, gene expression and DNA methylation data of the gene families in specific inflammatory diseases.

## Methods

**Fig. 1:**
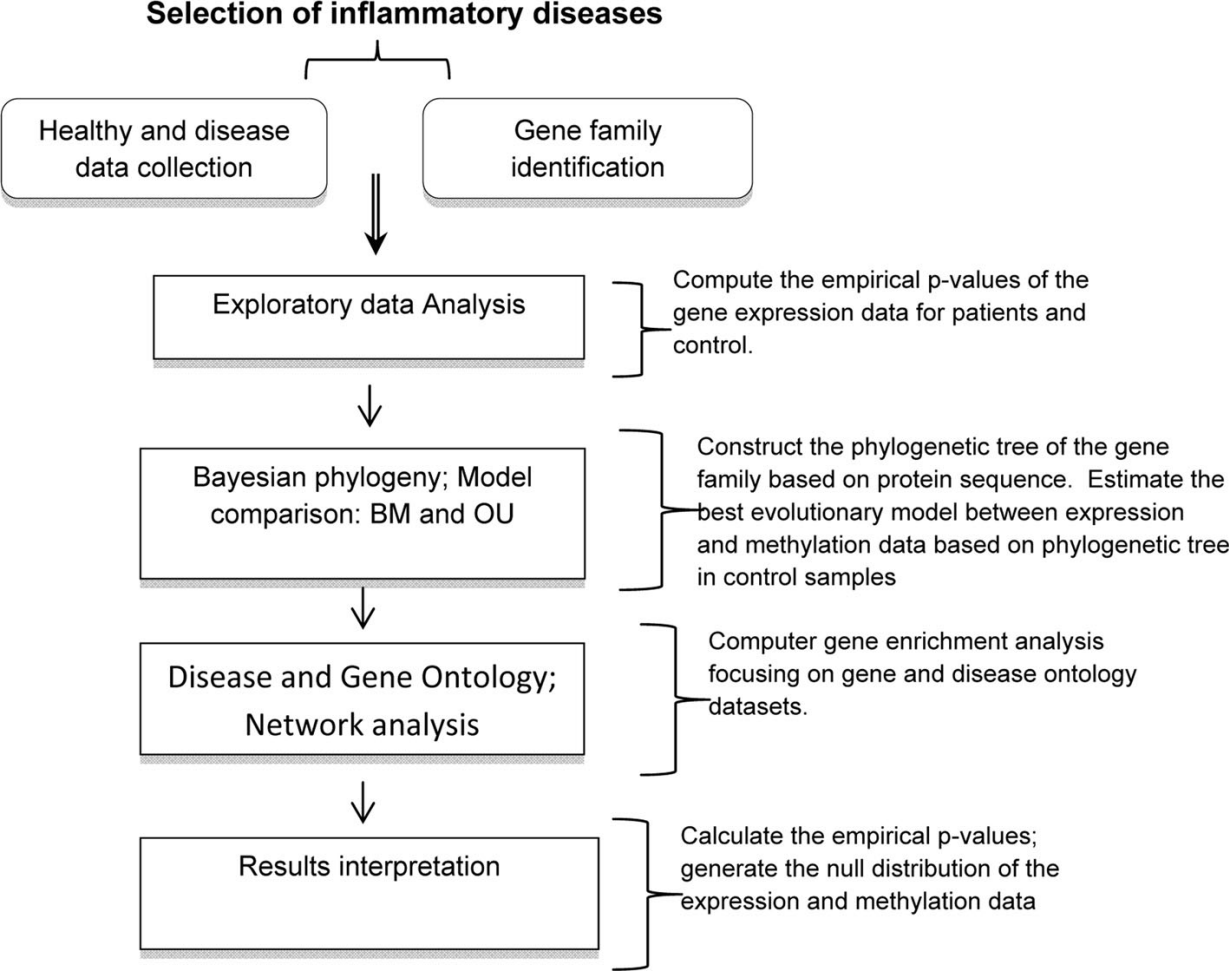
The flowchart of the methodology. To identify the significant members in a gene family which are significantly associated with a disease, the input data include the protein sequences of members of the gene family and the case–control gene expression and DNA methylation data of the gene family in the disease

The methodology of this work follows a two-step procedure: Firstly, we analyse case-control gene expression data of a number of inflammatory diseases, focusing on signalling factors and receptors. In the second step, we select the gene families including genes with statistical significant *p*-values in the first step focusing on specific inflammation related diseases for which both gene expression and DNA methylation data are available. We use an Ornstein–Uhlenbeck phylogenetic approach to identify disease associated genes by integrating the gene expression and DNA methylation data. On the basis of the identified genes, we explore the correlations among the inflammation related diseases. The flowchart of the whole analysis is shown in Fig. 1.

### Step 1: Genome–wide comparison of gene expression in inflammatory diseases

Recent research has increasingly demonstrated that many seemingly dissimilar diseases have common molecular mechanisms. Diseases are more likely to be comorbid if they share pathways. Exploring relations between genes and diseases at the molecular level could greatly facilitate our understanding of pathogenesis, and eventually lead to better diagnosis and treatment. Some diseases have a direct positive association between them while other diseases may have indirect positive associations among them through biological pathways. The analysis of pathway–disease associations, in addition to gene–disease associations, could be used to clarify the molecular mechanisms of a disease.

We take advantage of the large number of molecular measurements from experimental results that are now publicly available and identify commonly implicated genes across different pathologies and deliberately varied experimental conditions. We propose the application of a gene expression based genome–wide association study (eGWAS) to evaluate the statistical significance of the differential expression for each gene across a large number of case and control microarray experiments of human inflammatory diseases.

#### Gene expression data for Step 1

We collect a large ensemble of gene expression data related to diseases that have frequent inflammation comorbidities from different cell types/tissues in patients and healthy people, including Type 1 Diabetes, Type 2 Diabetes, Rheumatoid arthritis, Osteoporosis, Osteopetrosis, Osteoarthritis, HIV infection, Osteomyelitis, Measles, Paget’s disease, Periodontitis, Renal disorder, Osteosarcoma, Breast cancer and Multiple myeloma. The raw microarray gene expression data are downloaded from the Gene Expression Omnibus (GEO) (see Additional file 1).

#### Evaluation of differentially expressed genes

Using the case-control gene expression data, we evaluate the significance of differential expression of each gene between healthy and disease samples using a linear model based statistical method. Differentially expressed genes are selected as genes with significant *p*-values (e.g. p <0.05) in different diseases. In particular we focus on genes that code for extracellular signalling molecules (including receptors) as they are linked to metabolic physiological flexibility. The normalisation procedures and statistical analysis are implemented in R by using Bioconductor R packages [27].

For each raw microarray gene expression dataset, the background correction and normalization is performed by using the PLIER algorithm [28]. The PLIER algorithm uses a probe affinity parameter, which represents the strength of a signal produced at a specific concentration for a given probe. The error model employed by PLIER assumes that the error is proportional to the observed intensity, rather than to the background–subtracted intensity using the following error function:

1 $$ \epsilon_{ij}=\frac{\hat{\mu}_{ij}/{pm}_{ij} + \sqrt{\left(\hat{\mu}_{ij}/{pm}_{ij}\right)^{2} + 4\left({mm}_{ij}/{pm}_{ij}\right)}}{2},  $$

where, *μ*_*ij*_ is the binding level of probe *i* on array *j*, *pm*_*ij*_ is a perfect match and *mm*_*ij*_ is a mismatch probe.

Then, we sort out the genes with consistently highly differentially expressed between case and control samples using the following linear model:

2 $$ y_{ik}=\alpha_{k}+\epsilon_{ik} \;\; i = 1, 2,....,n_{k}, \;\; k = 1, 2,  $$

where *k* indicates the patient type and *i* the individual samples. For every gene g, we define the rank consistency score *S*(*g*;*r*) as the normalized maximal rank of this gene among all the patients samples, i.e.,

3 $$ S(g;r)={max}_{1ky} R_{k}(g)/N.  $$

### Step 2: Phylogenetic–based multi–omic analysis of gene families through an Ornstein–Uhlenbeck model

Gene families have extraordinary importance in elucidating genome dynamics, which combine the study of diseases at the pathway level with the evolutionary mutational divergence and selection trajectories. The evolutionary information is contained in a phylogenetic tree consisting of all the members of a gene family connected through the similarities of their sequences. The effects of phylogenetic relationships on observed phenotype data has been studied for a long time in evolutionary biology under the generic name of phylogenetic comparative methods. These methods assume a continuous time, continuous (or discrete for discrete phenotypes) space stochastic process for the phenotype and allow it to evolve on top of the phylogenetic tree. At speciation times the process splits into two independent copies which evolve along the branches. Then the process parameters can be inferred based on the law of the values at the tips (the contemporary species). Gene families may provide clues for identifying genes that are involved in particular diseases, e.g., chemokine receptor, TGF– *β* and TNF gene families play important roles in inflammatory diseases and cancers [29–35]. However, the coordinative functional relationships between members of the gene family are still unknown. Studying the dynamic regulation mechanisms will help understanding the genesis of the diseases and improving the effective drug discovery [36]. Because of the complexly structured interaction between the process of evolutionary functional divergence of the gene family members and the process of pathway proximity of some members, it is important to model together the two processes by using all the available multi–omic information such as epigenetic modification and gene expression. The multi–omic information could help identifying the trajectory between healthy and disease condition. For instance epigenetic variability may drive phenotypic selection on a much shorter timescale than mutation.

We can use an ecological analogy to describe together the healthy and disease conditions with their omic information. The mutational (generated by epigenetic modification and gene expression changes) and natural selection pressure could be modeled by an Ornstein–Uhlenbeck (OU) model acting on a landscape. The OU model is frequently used in physics to model an overdamped Brownian harmonic oscillator — that is, the stochastic variation from a normal state with no persistence of the rate of change — opposed by a stronger restoring force towards the equilibrium point. In our system, stochastic changes in multi–omic represent a restoring force constraining the patient in its normal state. A possible visualisation of healthy and disease states is the landscape analogy described by Waddington where the multi–omic information determines the walls of a valley that traps a rolling ball (the condition of the patient). The walls act as restoring forces (representing the natural selection). The multi–omic information may provide a potential mechanism for control of the level of the phenotypic variation (which is represented by the slope of the valley walls). In diseases the multi–omic deregulation would change the valley, altering the balance of regulation that maintained the stable multi–omic signature in the face of noise. This could be the result of repeated restructuring of the multi–omic landscape through inflammatory condition. Clearly different diseases have specific multi–omic structure requirements, i.e. valleys.

The multi–omic OU could be considered within a phylogenetic framework [37–41], which is biologically motivated by the ideas of adaptation, selection, stasis. The mean phenotype of the species is expected to exhibit small oscillations around an optimal state defined by the environment where it lives in stasis [42]. If the environment changes, the optimal state will be affected, which will lead to rapid evolution of species towards the new optimum.

The concepts from the evolutionary biology have been recently used in the study of genes [43, 44]. Furthermore, Bartoszek and Lio’ [45] used a branching Brownian motion (BM) process, implemented in the mvSLOUCH package [37], to distinguish between competing phylogenetic trees for bacterial species. The aim of this work is to find the associations between diseases and the genes in a gene family. Phylogenetic comparative methods can provide us with a probability law that takes into account the phylogeny. We can then estimate the expected variability at the level of tips and test that if a certain gene lies outside the null distribution of healthy cases.

**Fig. 2:**
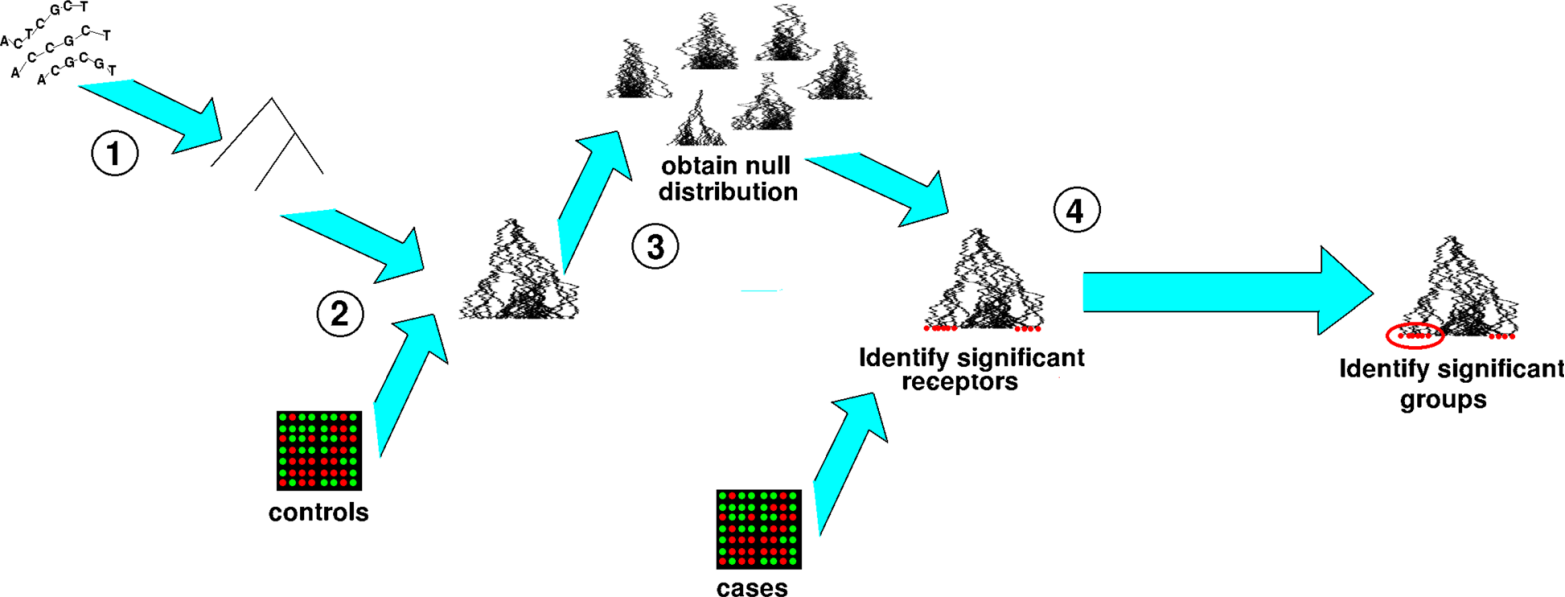
The scheme of the phylogenetic steps. 1) Construct the phylogenetic tree of the gene family based on the protein sequences; 2) Estimate the best evolutionary models between expression and methylation data based on the phylogenetic tree in control samples; 3) Generate the null distributions of the expression and methylation data for the gene family following the best evolutionary model; 4) Calculate the empirical *p*-values of the expression and methylation levels for each member of the gene family in patient samples and select the significant ones

In terms of the phylogenetic trees connecting the genes, more recently diverged genes should show similar expression levels due to the common descent. Moreover, the association with a disease of one member in the gene family suggests that closely related genes will have a higher chance of sharing some of this relationship than more distant ones. Recent studies found that DNA methylation plays a regulatory roles on gene expression [46]. The methylation and expression levels of genes usually exhibit dependencies in human diseases such as cancers [47]. Recent studied of DNA methylation data have provided evidences of different patterns of changes existing at promoter and gene body levels [48, 49]. Therefore, in this paper, we study the roles of gene promoter methylation and gene body methylation vs. gene expression respectively constrained by the phylogenetic information. The proposed methodology is a generalized method and could easily accommodate multiple epigenetic features, constrained only by the computational capabilities of available software. In the following sections, we provide the theoretical basis of the methodology. The flowchart of Step 2 is shown in Fig. 2.

#### DNA methylation and gene expression data for Step 2

In Step 2, we focus on nine inflammation related diseases, Allergy, Asthma, Ulcerative Colitis (Colitis), Crohns’ Disease (Crohn), Rheumatoid Arthritis (RA), Chronic Fatigue Syndrome (CFS), Systemic Lupus Erythematosus (SLE), Type 2 Diabetes (T2D) and Colon Cancer, which have both gene expression and DNA methylation data (see Additional file 2). The methylation (promoter and gene body) data of all the diseases were measured by Illumina Infinium HumanMethylation450 (450K) BeadChip. The downloaded data have already been prepocessed and normalized into probe level. To convert the data from probe level to gene level, we map the probes to Entrez GeneID following the annotation files of the microarray platforms. Probes mapped to multiple genes are deleted. For the gene expression data, the average of the values of the probes corresponding to the same gene, is calculated as the expression value for the gene. For the methylation data, we keep the probes which are mapped to the promoter (TSS200) and gene body regions, and take the average of methylation values of the probes corresponding to the two regions respectively as the two methylation features for each gene.

#### Construction of phylogenetic correlation models based on expression and methylation of a gene family

We assume that the mean expression and methylation levels (denoted by $\vec {X}(t)$) evolve on a phylogeny following a multivariate *k*–dimensional Ornstein–Uhlenbeck process

4 $$ \vec{X}(t) = -\mathbf{A}\left(\vec{X}(t) - \vec{\theta}\right)dt + \mathbf{\Sigma}d\vec{W}(t),  $$

where $\vec {W}(t)$ is a multivariate standard Wiener process. The process parameters are the matrices **A** (can be in particular **0** or have zero rows), **Σ** and the vector $\vec {\theta }$. The process is multivariate normal with mean and variance equalling

5 $$ \begin{aligned} \mathbf{E}\left[\vec{X}\right](t) &= e^{-\mathbf{A}t} \vec{X}(0) + \left(\mathbf{I} - e^{-\mathbf{A}t} \right) \vec{\theta}\\ & \quad \mathbf{Var}\left[\vec{X}\right](t)= \int\limits_{0}^{t}e^{-\mathbf{A}s} \mathbf{\Sigma} \mathbf{\Sigma}^{T} e^{-\mathbf{A}^{T}s} ds. \end{aligned}  $$

If all the eigenvalues of **A** have positive real part, then the process converges weakly to its stationary normal distribution with mean equalling $\vec {\theta }$ and covariance matrix equalling

$$\mathbf{P}\left(\left[ \frac{1}{\lambda_{i} + \lambda_{j}} \right]_{1 \le i,j \le k} \odot \mathbf{P}^{-1}\boldsymbol{\Sigma}\boldsymbol{\Sigma}^{T}\mathbf{P}^{-T} \right)\mathbf{P}^{T}, $$

where ⊙ is the Hadamard product, *λ*_*i*_s and **P** are respectively the eigenvalues and eigenvectors of **A**.

The mvSLOUCH R package [37] is used to estimate the parameters of the process for modelling expression, methylation and the gene level evolution. We use the mvSLOUCH package for our analysis because currently it offers the widest choice of possible models for the multivariate trait. Importantly for us it allows for some of the traits to evolve as Brownian motion (i.e. neutrally) while for the others to be under selective pressure to track changes in the “Brownian ones”. This wider spectrum of tested models (the wrapper function running the analysis tries out a whole collection of parametrizations of the OU equation, this wrapper function has been incorporated into the public interface of mvSLOUCH and its functionality may be exploited by calling mvSLOUCH::estimate.evolutionary.model()) allows for better exploration of the parameter space but moreover facilitates interpretation—how do the different variables interact with each other. We compare (by AIC _*c*_) Brownian motion (**A**=**0**), stationary and non–stationary OU models, and specify that certain variables evolve marginally as Brownian motion (equivalent to setting in **A** rows corresponding to them to **0**).

The parameters of the OU process have very sophisticated interpretations in the evolutionary biology field [41, 50]. In this work, it is assumed that in a constant environment (e.g. healthy person), called selective regime in evolutionary terminology, the expression and methylation levels should exhibit stationary oscillations around an optimal state. This stasis situation can be modelled by an (multivariate) OU process. If a gene property is associated with a disease, then its levels will be significantly out of the band predicted by the stationary oscillations.

The values associated with each gene are the means and variances of the expression and methylation for the populations (case or control individuals) under study. There is always a natural variation inside a population, and the expression and methylation patterns are very variable. Ignoring this variation could lead to spurious conclusions and therefore it should be taken into account. The variation is expected to be dominated by the specific conditions which the individual lived in and hence not to exhibit an evolutionary history. The estimate of this variation is the sample variance for the expression or methylation of the gene. This is a standard procedure in phylogenetic comparative methods and the variation appears in any downstream analysis as uncorrelated measurement error [37, 51, 52] and it is added to the diagonal of the between–gene family member–between–traits variance–covariance matrix.

#### Detection of disease-associated members of a gene family

Genes, whose expression levels in disease samples significantly deviate from the optimal evolutionary model (gene expression vs. promoter/body methylation) estimated based on the healthy samples, are defined as dysregulated expressed genes (DEGs). Similarly, genes, whose promoter/body methylation levels in disease samples significantly deviate from the corresponding optimal evolutionary model, are defined as dysregulated methylated genes (DMGs).

The identification of DEGs and DMGs requires the estimation of the parameters of the stochastic process generating by the control levels. On the basis of the phylogeny, we simulate 200,000 independent evolutions of expression and methylation levels under the law of this process with the estimated parameters, which gives us the null distribution for the levels that includes the ancestral dependencies between the different genes. Then, the empirical *p*-values are calculated to assess if a case measurement for a gene is significantly different from its control counterpart. After simulating the process for each tip we take the difference between the simulated control values and true control values. Then, the *p*-value is calculated by comparing the observed difference between cases and controls to the null distribution of the difference between simulated and true control values.

The genes with significant *p*-values (e.g., *p* <0.05) are selected as DEGs and DMGs which are considered significantly associated with a disease. Although this approach alone does not really guard us against multiple testing issues as we look at individual *p*-values [53], our aim is to build an overall network by building up on all the evidences from the families. Therefore, a gene family member with a marginal *p*-value (e.g., around 0.05) could be considered interesting or suggestive if it has pathway connections with other genes with significant *p*-values belonging to other families. The significance of a gene suggests the strong association with the genesis of the disease. Because of the high correlation between the members of the family induced by the shared ancestry and phylogenetic inertia, a too stringent approach at single gene family could vanish the opportunity of evaluating the evidence synthesis across the overall gene family network through Gene Ontology.

#### Construction of functional associations between diseases

If a gene is associated with two different diseases, it is likely that the two diseases share the similar functional mechanisms involving this common gene at molecular level. Consequently, the functional consistence between two diseases can be evaluated based on the overlapping of the corresponding associated genes. The genes involved in the same biological process usually exhibit high consistence in function. Here we use the Gene Ontology (GO) semantic similarity measure proposed by [54] to evaluate the functional similarities of the associated genes for two diseases.

The semantic similarity measure is an information content which takes into account the hierarchical structure of GO. It is calculated by the frequencies of two GO terms and that of their closest common ancestor term in the directed acyclic graph (DAG) of GO. The information content of a GO term is calculated by the negative log probability of the genes occurring in the GO term and all of its children terms against the total genes annotated in GO. The frequency of a GO term *t* is computed as:

6 $$ p(t) = \frac{n_{t^{\prime}}}{N}, \qquad t^{\prime} \in {\{t, \text{children of t}\}},  $$

where $\phantom {\dot {i}\!}n_{t^{\prime }}$ is the number of genes annotated in term t and all of its children terms, and *N* is the number of total genes annotated in GO. The information content (IC) of GO term *t* is defined as:

7 $$ IC(t) = -\log(p(t)).  $$

Because a GO term could have multiple parents in the DAG, two terms can share parents by multiple paths. The similarity between two GO terms is calculated based on the information content of their closest common ancestor term which is also called the most informative common ancestor (MICA). As proposed by [54], the semantic similarity between GO terms *t*_1_ and *t*_2_ with the most informative common ancestor term MICA is computed as:

8 $$  sim(t_{1},t_{2}) = \frac{2IC(MICA)(1-p(MICA))}{IC(t_{1})+IC(t_{2})}.  $$

The functional similarity between two genes is calculated based on their corresponding annotated GO terms. Given two GO terms sets *GO*_1_={*go*_11_,*go*_12_,...,*go*_1*n*_} and *GO*_2_={*go*_21_,*go*_22_,...,*go*_2*n*_} annotated by gene *g*_1_ and *g*_2_ respectively, the similarities matrix between *GO*_1_ terms and *GO*_2_ terms is computed following Eq. (9). The similarity score between the two genes is calculated as the average of all maximum similarities on each row and column of the GO terms similarity matrix:

9 $$  {}sim(g_{1}, g_{2}) \! = \! \frac{ \! \sum\limits_{i=1}^{m} \! \max \limits_{1 \leq j \leq n} \! sim({go}_{1i},{go}_{2j}) \! +\! \! \sum\limits_{j=1}^{n} \! \!\max \limits_{1 \leq i \leq m} \! \!sim({go}_{1i},{go}_{2j})}{m+n} \!.  $$

## Results

### Genome-wide analysis of gene expression in human inflammatory diseases reveals several interested signalling gene families

Following the Step 1 analysis procedure that introduced in the section of Methods, a number of significantly differentially expressed genes are identified in the selected inflammatory diseases (see Additional file 3). The significant differential expression of these genes in the patients compared with the healthy samples suggests strong associations between the genes and the inflammatory diseases. The genes are involved in several important signalling gene families related with inflammation such as the chemokine receptor family, the tumor necrosis factor (TNF) receptor family, the transforming growth factor beta (TGF– *β*) family and the interleukin 17 (IL–17) family. The comparative analysis of the selected inflammatory diseases shows that the TNF receptor family and the TGF– *β* gene family are more differentially expressed between healthy and disease samples. There are more members in these two gene families exhibiting significant *p*-values than the chemokine receptor family. The signalling molecules of IL–17 family are represented by Rel A, B, TRAF, NFkB1 and NFkB2 related gene members. Their enrichment presence in the pool of disrupted genes in all diseases highlights the crosstalk with the NF–kappaB signaling pathway. The TGF– *β* family is involved in most of the considered diseases, e.g., TGFB1 in Osteoporosis, TGFB3 in Rheumatoid arthritis, Osteoarthritis and Multiple myeloma, TGFBR2 in HIV, Osteomyelitis and Measles, BMP in Breast cancer and Osteosarcoma, and BMP3 in Periodontitis. The TNF receptor family is involved in many of the selected inflammatory diseases, e.g., TNFRSF10B in Osteoporosis, Osteomyelitis and periodontitis, TNFRSF11B and TNFSF13B in Osteopetrosis and Periodontitis, and TNFIP6/8 in Osteomyelitis.

### Phylogenetic–based multi–omic analysis suggests potential disease associated genes of four signalling gene families: the chemokine receptor family, the TNF receptor family, the TGF– *β* family and the IL–17 family

#### Case study of the chemokine receptor family

Chemokine receptors belong to the large G–coupled protein receptors family and are abundantly expressed in a variety of immune cells, playing a crucial role in the immune system by binding with chemokines [36]. Accumulating evidence has provided insight into the importance of chemokine and chemokine receptors in various diseases including cancers, HIV and inflammatory diseases [55]. For example, the chemokine receptors CXCR4 and CCR7 have been found to be involved in breast cancer metastasis [56], and both CXCR4 and CCR5 have been successfully used as drug targets for haematopoietic stem cell mobilization and HIV inhibition [57]. Despite the growing effort in developing drugs targeting chemokine receptors, there has been limited success in clinical trials concerning inflammatory diseases. The effects of biased signalling mechanisms at receptor level for the fine–tuning of the immune system [36] have not been clearly understood yet. Growing evidence of the biased signalling of the chemokine gene family implies that different chemokines activate specific signalling pathways via binding to the corresponding receptors in different inflammatory diseases. Studying this dynamic regulation mechanisms will help understanding the genesis of the diseases and improving drug discovery. Here, we apply the proposed phylogenetic–based multi–omic method on the chemokine receptor family to detect the members of this family which are significantly associated with different inflammatory diseases.

**Fig. 3:**
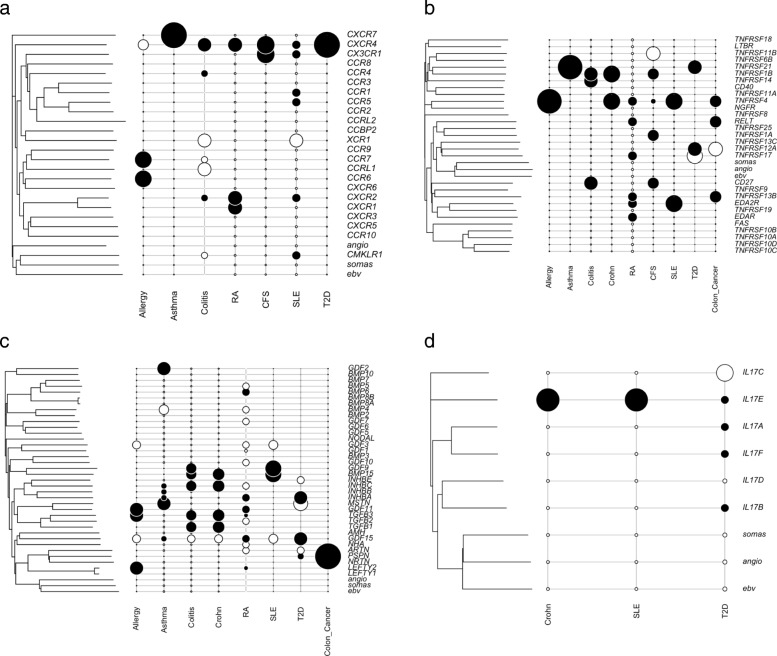
Phylogenetic correlation analysis for gene families: **a** the chemokine receptor family; **b** the TNF receptor family; **c** the TGF– *β* family; **d** the IL–17 family. In each figure, the phylogenetic tree for the protein sequences of the gene family constructed using neighbour–joining is shown on the left side. The sequences EBV (human EBV-induced G protein-coupled receptor), angio (human type-1 angiotensin II receptor isoform) and somas (human somatostatin receptor) are considered as outgroups. The scale bar refers to the branch lengths, measured in expected numbers of amino acid replacements per site. The significant association between diseases and genes of the gene family is shown on the right side. Large black circles represent up–regulated expression; small black circles represent down–regulated expression; large white circles represent up–regulated (gene body AND promoter) methylation; small white circles represent down–regulated methylation

Using the multivariate OU framework, the optimal correlation models between gene expression and promoter/body methylation data taking account of phylogeny information for chemokine receptors are estimated in controls, which are shown in Table 1. The optimal correlation models for gene expression vs. promoter methylation are almost the same with the ones for gene expression vs. body methylation, which mainly follow a bivariate OU model (OUOU) in eight disease except Colitis in which the best correlation model is a BM model. The disease–associated chemokine receptors with significant dysregulation in expression or methylation are shown in Table 2. The expression levels or the methylation levels in the gene body regions of the significant chemokine receptors in the patients of Allergy, Asthma and Colitis do not follow the estimated correlation models in the corresponding control samples, which suggests that these significant chemokine receptors may be involved in the epigenetic regulation mechanisms during the genesis of the diseases. There is a preponderance of the gene expression effects over the gene body and promoter methylation and a preponderance of gene body over promoter methylation. The phylogenetic correlation between the multi–omic information, diseases and genes is shown in Fig. 3a.

**Table 1:** Optimal evolutionary models of DNA methylation and gene expression for the four gene families in controls (promoter methylation vs. gene expression ∣ gene body methylation vs. gene expression)

Disease	Chemokine receptor	TNF receptor	TGF– *β*	IL17
Allergy	OUOU ∣ OUOU	OUOU ∣ OUOU	OUOU ∣ OUOU	OUOU ∣ OUOU
Asthma	OUOU ∣ OUOU	OUOU ∣ OUOU	OUOU ∣ OUOU	OUOU ∣ OUOU
CFS	OUOU ∣ OUOU	OUOU ∣ OUOU	OUOU ∣ OUOU	OUOU ∣ OUOU
Colitis	BM ∣ BM	OUOU ∣ OUOU	OUOU ∣ OUOU	OUOU ∣ OUOU
Colon Cancer	OUOU ∣ OUOU	OUOU ∣ BM	OUOU ∣ OUOU	OUOU ∣ OUOU
Crohn	OUOU ∣ OUOU	BM ∣ OUOU	OUOU ∣ OUOU	OUOU ∣ OUOU
RA	OUBM ∣ OUOU	OUOU ∣ OUOU	OUOU ∣ OUOU	BM ∣ OUOU
SLE	OUOU ∣ OUOU	OUOU ∣ OUOU	OUOU ∣ OUOU	OUOU ∣ OUOU
T2D	OUOU ∣ OUOU	OUOU ∣ OUOU	OUOU ∣ OUOU	BM ∣ BM

OUOU: bivariate OU model, both methylation and expression follow OU model; BM: Brownian Motion model, both methylation and expression follow BM model; OUBM: methylation and expression follow different models

**Table 2:** Significant disease associated genes in the chemokine receptor family

Disease	Up–regulated DEG	Down–regulated DEG	Up–regulated DMG	Down–regulated DMG
Allergy	CCR6, **CCR7**	—	—	*CXCR4*
Asthma	**CXCR7**	—	—	—
CFS	CXCR4, CX3CR1	—	—	—
Colitis	**CXCR4**	**XCR1**, **CCRL1**	*CCR4*	*CCR7,CMKLR1*
Colon Cancer	—	—	—	—
Crohn	—	—	—	—
RA	CXCR1, CXCR2, CXCR4	—	—	—
SLE	CCR5, CMKLR1, CXCR2, CXCR4, CX3CR1, CCR1	XCR1	—	—
T2D	CXCR4	—	—	CXCR4

DEG stands for dysregulated expressed genes and DMG stands for dysregulated methylated genes. Genes in bold represent significant dysregulations according to both promoter methylation model and gene body methylation model; Genes in italic represent significant dysregulations according to promoter methylation model; Genes in normal fonts represent significant dysregulations according to gene body methylation model

#### Case study of the TNF receptor family

The TNF gene family includes 29 receptors which are trimeric cytokine receptors that bind tumor necrosis factors (TNFs). These receptors are important in determining the response outcome (e.g. apoptosis, inflammation), which suggests their potential roles associated with diseases. The phylogenetic correlation between the multi–omic information, diseases and genes is shown in Fig. 3b. The correlation between promoter/body methylation and expression of the TNF receptor family follows the bivariate OU model in most diseases except Colon Cancer and Crohn. As shown in Table 1, the optimal model for gene expression vs. body methylation in Colon Cancer follows the BM model and the optimal model for gene expression vs. promoter methylation in Crohn follows the BM model. The TNF receptors which are associated with the diseases are shown in Table 3. Many members in TNF receptor family show significant dysregulation in expression or methylation in CFS, Colitis, Colon Cancer, RA and T2D. The disruption at the level of promoters seems more important than the disruption at gene body level.

**Table 3:** Significant disease associated genes in the TNF receptor family

Disease	Up–regulated DEG	Down–regulated DEG	Up–regulated DMG	Down–regulated DMG
Allergy	—	—	*TNFRSF4*	—
Asthma	*TNFRSF21*	—	—	—
CFS	TNFRSF1A, CD27, TNFRSF1B	TNFRSF11B	*TNFRSF4*	—
Colitis	TNFRSF14, CD27, TNFRSF1B	—	—	—
Colon Cancer	—	—	RELT, *TNFRSF4*, *TNFRSF13B*	TNFRSF12A
Crohn	*TNFRSF14*, *TNFSRF1B*	—	—	—
RA	—	—	RELT, *TNFSRF4*, *TNFRSF17*, *TNFRSF13B*, *EDA2R*, *EDAR*	—
SLE	*TNFRSF1B*	—	*TNFRSF4*, *EDA2R*	—
T2D	**TNFRSF21**, **TNFRSF12A**	**TNFRSF17**	—	—

DEG stands for dysregulated expressed genes and DMG stands for dysregulated methylated genes. Genes in bold represent significant dysregulations according to both promoter methylation model and gene body methylation model; Genes in italic represent significant dysregulations according to promoter methylation model; Genes in normal fonts represent significant dysregulations according to gene body methylation model

#### Case study of the TGF– *β* family

The transforming growth factor beta (TGF– *β*) family plays key roles in cell proliferation and differentiation, and other important biological processes [31]. Members of the TGF– *β* family are synthesized as prepropeptide precursors that are processed into mature, biologically active homodimers or heterodimers, which activate serine/threonine kinase receptors. Scientific evidence shows that the TGF– *β* proteins are involved in the genesis of several diseases such as immunity, cancer, bronchial asthma, lung fibrosis, heart disease, diabetes, Parkinson’s disease, and AIDS [58]. The phylogenetic correlation between the multi–omic information, diseases and 36 genes of TGF– *β* family is shown in Fig. 3c. As shown in Table 1, the correlations between promoter/body methylation and expression of the TGF– *β* family follow a bivariate OU model in all situations. The significant genes in the TGF– *β* family which are associated with the diseases are shown in Table 4. There are several members in TGF– *β* family that are dysregulated significantly in expression (in particular) or methylation in most diseases, but in CFS there is none and in Colon Cancer there is only one gene significantly up–regulated in gene body methylation. There is a good agreement (bold in Table 4) between gene body and promoter methylation relationship with respect to gene expression.

**Table 4:** Significant disease associated genes in the TGF– *β* family

Disease	Up–regulated DEG	Down–regulated DEG	Up–regulated DMG	Down–regulated DMG
Allergy	**GDF11**, LEFTY2, **TGFB3**	—	—	GDF3, GDF15
Asthma	**GDF2**, **GDF15**, **MSTN**, **INHBA**, **INHBB**, **INHBC**	**BMP4**	**GDF2**, *GDF9*, BMP15, MSTN, PSPN	—
CFS	—	—	—	—
Colitis	**TGFB1**, **TGFB3**, **BMP15**, **INHBC**, **GDF9**	—	—	GDF15
Colon Cancer	—	—	PSPN	—
Crohn	**TGFB1**, **TGFB3**, **BMP15**, **INHBC**	—	—	GDF15
RA	**GDF11**, GDF15, INHBA, **BMP6**	GDF10, TGFB2, GDF3, ARTN, BMP4, GDF7, BMP5, INHBC, INHA	LEFTY2, TGFB3	GDF1
SLE	BMP15, GDF9	—	—	GDF3, GDF15
T2D	GDF15, INHBA	MSTN	PSPN	ARTN, INHBE

DEG stands for dysregulated expressed genes and DMG stands for dysregulated methylated genes. Genes in bold represent significant dysregulations according to both promoter methylation model and gene body methylation model; Genes in italic represent significant dysregulations according to promoter methylation model; Genes in normal fonts represent significant dysregulations according to gene body methylation model

#### Case study of the IL–17 family

The interleukin 17 (IL–17) family plays a crucial role in host defence against microbial organisms and in the genesis of proinflammatory diseases. IL–17 is commonly associated with allergic responses. IL–17 induces the production of many other cytokines (such as IL–6, G–CSF, GM–CSF, IL–1 *β*, TGF– *β*, TNF– *α*), chemokines (including IL–8, GRO– *α*, and MCP–1), and prostaglandins (e.g., PGE2) from many cell types (fibroblasts, endothelial cells, epithelial cells, keratinocytes, and macrophages). TGF– *β* and chemokines (IL–6) drive the production IL–17 cytokines in immunity and inflammation [59–61]. The phylogenetic correlation between the multi–omic information, diseases and genes of the IL–17 family is shown in Fig. 3d. Here, we also applied the proposed methods to the IL–17 gene family. The correlation between promoter/body methylation and expression of the IL–17 family follows the bivariate OU model in most diseases except T2D and RA in which the best correlation models follow the BM model (Table 1). The significant genes in the IL–17 family which are associated with the diseases are shown in Table 5. There are only significantly dysregulated genes in CFS, Crohn, SLE and T2D.

**Table 5:** Significant disease associated genes in the IL–17 family

Disease	Up–regulated DEG	Down–regulated DEG	Up–regulated DMG	Down–regulated DMG
Allergy	—	—	—	—
Asthma	—	—	—	—
CFS	—	IL17A	—	—
Colitis	—	—	—	—
Colon Cancer	—	—	—	—
Crohn	**IL17E**	—	—	—
RA	—	—	—	—
SLE	**IL17E**	—	—	—
T2D	—	—	**IL17F**, *IL17A***IL17B***IL17E*	*IL17C*

DEG stands for dysregulated expressed genes and DMG stands for dysregulated methylated genes. Genes in bold represent significant dysregulations according to both promoter methylation model and gene body methylation model; Genes in italic represent significant dysregulations according to promoter methylation model; Genes in normal fonts represent significant dysregulations according to gene body methylation model

#### Curated evidence for the identified disease associated genes

We find a selection of evidences from biomedical literature, which prove the involvement of many identified genes in the genesis of the corresponding diseases (Table 6). Although no clear evidence has been reported for the other identified genes, there are some interesting clues. For example, ARTN has not been proved to be associated with T2D yet, but it is associated with Hirschsprung’s disease 1 and Parkinson disease, late–onset. It plays an important role in pathways related to developmental biology and Interleukin receptor SHC signaling, and strong attractant of gut hematopoietic cells thus promotes the formation Peyers patch–like structures [62]. Although there are no report on the involvement of the GDF1 in RA, it is associated to transposition of great arteries, dextro-looped 3 and right atrial isomerism [63]. There are no report on the involvement of GDF9 in Crohn, but mutations in GDF9 can result in sterility and lower ovulation rate [64].

**Table 6:** Literature evidence for the identified disease associated genes in the four signalling gene families

Disease	Disease genes with evidence
Allergy	CCR7 [70]; TNFRSF4 [71]; CCR6 [72]
Asthma	CXCR7 [73]
CFS	TNFRSF4 [74]; TNFRSF1A [75]
Colitis	TGFB1 [76]; TGFB3 [77]; XCR1 [78]; CCR4 [79]; CKMLR1 [80]; CXCR2 [81]; CXCR4 [82]; CD27 [83]; CCR7 [83]; TNFRSF1B [84]
Crohn	GDF15 [85]; TNFRSF1B [86]; INHBC [87]; TGFB3 [77]; IL17E [88]; TGFB1 [76]
RA	GDF15 [89]; GDF1 [90]; GDF11 [91]; EDAR [92]; CXCR1 [93]; Relt [94]; TNFRSF13b [95]; TGFB2 [96]; TGFB3 [97]; CXCR2 [98]; BMP4 [99]; BMP5 [99]; BMP6 [100]; INHBA [101]
SLE	TNFRSF4 [102]; CX3CR1 [103]
T2D	GDF15 [104]; IL17A [105, 106]; IL17F [107]; ARTN [62]; INHBA [108]; CXCR4 [109, 110]; TNFSR17 [111]; TNFSR14 [112]; MSTN [113]; BMP4 [114]; TNFRSF21 [115]; TNFRSF12A [116]

### Phylogenetic–based multi–omic analysis of signalling gene families reveals functional associations between inflammation related diseases

#### Disease gene association networks

**Fig. 4:**
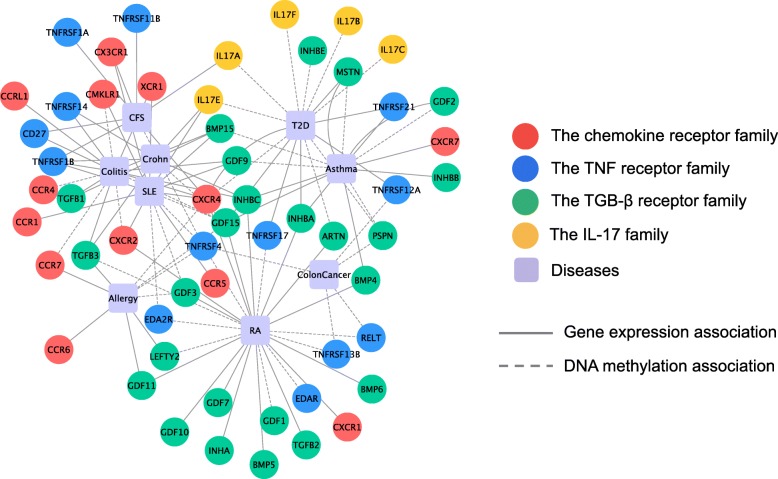
Gene–disease associations between the diseases and significant genes of the four families based on gene expression and methylation. Circle nodes represent genes and square nodes represent diseases. The abbreviations of the diseases are show in Additional file 2. Nodes represent genes and diseases. Edges between two nodes represent the associations between genes and diseases: solid lines for the associations in expression and dashed line for the associations in methylation

The gene–disease association network (Fig. 4) are constructed from the significant genes of the four gene families which are identified by the proposed multi–omic analysis. As shown in the network, the four signalling related gene families are prone to be associated with different diseases. For example, the chemokine receptor family may play important roles in Allergy, CFS, Colitis, RA, SLE and T2D, while the IL–17 gene family is probably related with T2D, CFS, Crohn and SLE. But the TGF– *β* and TNF receptor families are more likely to be involved in all the nine inflammation related diseases. The genes which link to multiple diseases in the network suggest the common molecular mechanisms for the diseases, which also provide clues for exploring the functional associations for disease comorbidities.

**Fig. 5:**
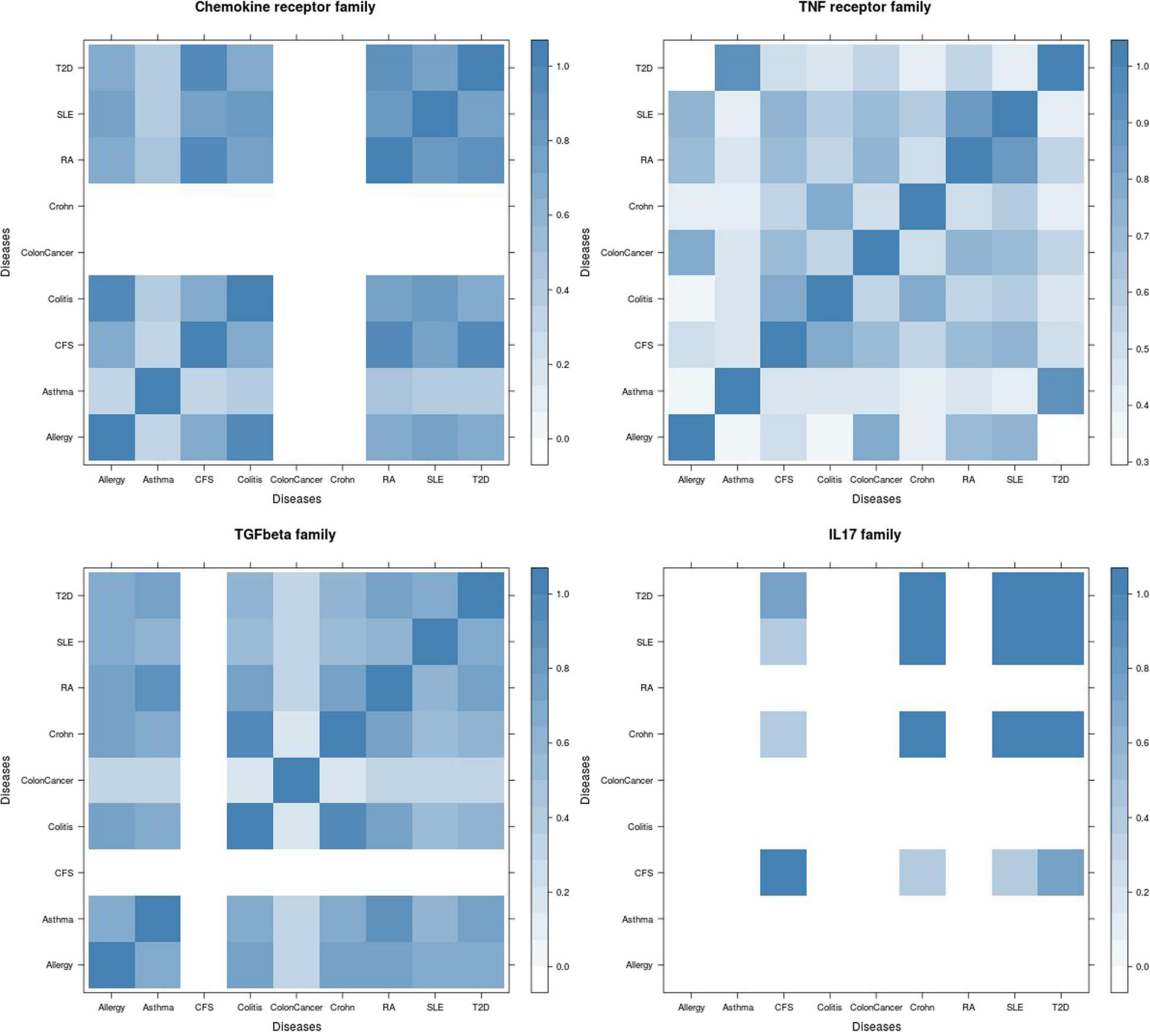
Functional consistence of diseases based on the significant genes of each gene family

To explore the functional consistence among the nine diseases on each gene family, we calculate the Gene Ontology semantic similarity between the genes which are significantly associated with the diseases. The functional similarity among the diseases on the four gene families are shown in Fig. 5. The strong similarity between two diseases suggests that the diseases are probably induced by the same disrupted biological pathways. For instance, the high functional consistency between RA, SLE and T2D in chemokine receptor family suggests that these diseases are more likely to involve similar functional mechanisms of the epigenetic regulations on the pathways related with chemokine receptors.

#### Inflammation map

**Fig. 6:**
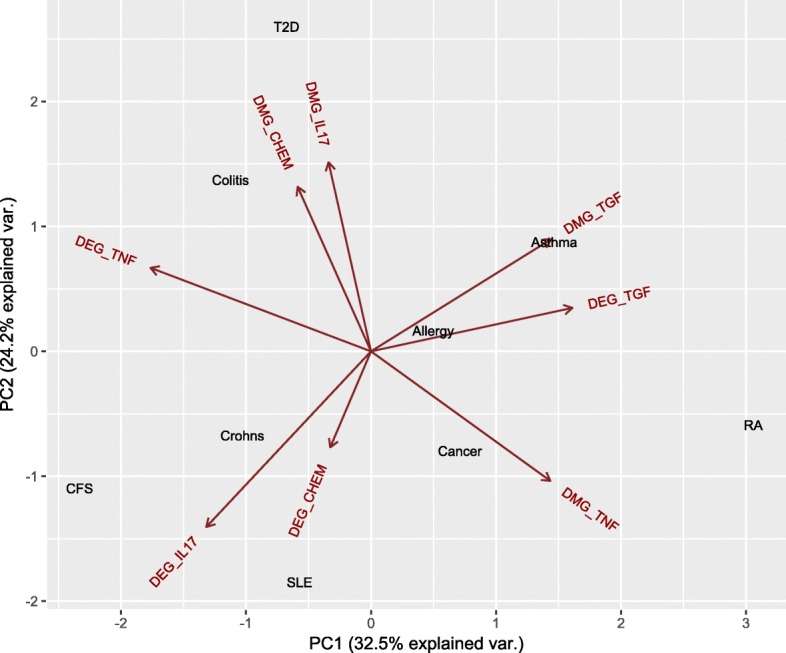
Principal component analysis (the first two principal components) of the matrix of disease–methylation and disease–expression associations. The figure shows relationships between diseases and dysregulation of gene expression and DNA methylation in the four signalling gene families

Central to inflammation pathology studies is the molecular analysis of inflammatory comorbidities and the comorbidity map which addresses the occurrence of different medical conditions or diseases, usually complex and often chronic ones, in the same patient. A meaningful way to summarise the relationship between diseases and multi–omic information is to compute the principal component analysis of the matrix that contains diseases and the numbers of changes in the methylation and gene expression in each of the four families. Figure 6 shows the first two principal components of the disease–methylation and disease–gene expression associations, in which the up-dysregulation and down-dysregulation in gene expression and promoter/body methylation are combined together.

## Discussion

The proposed method provides a tool to study the involvement of gene families in human disease by integrating gene expression, DNA methylation and gene sequences through a phylogenetic approach. The different models of adaptation (OU and BM) can be evaluated and compared based on the evolutionary relationship of the gene family by using gene expression and methylation data. The members of a gene family whose expression or methylation levels that do not follow the corresponding optimal evolutionary models are considered as the genes significantly associated with the diseases, which suggests the involvement of such genes in the epigenetic regulation mechanisms related to the genesis of diseases.

We believe that our proposed methodology provides a meaningful approach to compare the contribution of different omic data (DNA methylation and gene expression) and different genes within a family/group to a disease condition. The proposed methodology could be extended by integrating other omic data in the future. Currently, the methodology is primarily limited by the amount of multi–omic data (traits) that phylogenetic comparative methods can handle. Previously it was up to five or six traits, depending on the size of the phylogeny, or methods that calculated the whole between–species–between–traits variance–covariance matrix. However, right now there is tremendous progress in speeding up likelihood calculations for OU–based evolutionary models [65–68]. These new methods are based on either the three-point structure [67] or Felsenstein’s pruning algorithm [69], which allow for the likelihood to be evaluated in linear (in the number of tips) time and hence should make phylogenetic approaches a key multi-omic integration step, instead of a computational bottleneck. Thus, the new methods hold promise that it should be possible to analyse scores of traits for thousands of species in the nearby future. In addition, the proposed method for phylogenetic–based multi–omic analysis is limited to a single gene family because the hypothesis of the model assumes that the correlation between gene expression and DNA methylation of genes from a family is constrained by the evolutionary relationship of the gene family. But in practice, the roles of DNA methylation for gene expression regulation are complex and involve not only genes within the same family but possibly also genes from other functionally related gene families. In the future, the methodology could be extended to multiple gene families by taking into account the functional crosstalks between different families.

## Conclusions

We have performed a comparative study to explore the influence of signalling gene families in several inflammation related diseases. Firstly, we analyse gene expression in a collection of inflammatory diseases, which highlights the importance of gene families involved in extracellular signalling. In particular we have identified four families significantly associated with the inflammatory diseases, which includes the chemokine receptors family, the TNF receptor family, the TGF– *β* family and the IL–17 family. Then, in order to understand the roles these gene families in some specific inflammation related diseases, we propose a phylogenetic–based multi–omic method to study the correlations between gene expression and DNA methylation of the members of each gene family taking into account of their evolutionary relationships.

Applying the proposed method to four signalling gene families in nine inflammation related diseases, we identify a number of significant disease associated genes whose expression or methylation levels in the patients significantly deviate from the evolutionary models estimated from the control samples. Our results suggest that these families involve in different specific diseases. The chemokine receptor family may play important roles in Allergy, Asthma and Colitis, while the TNF receptor family may play key roles in CFS, Colitis, Colon Cancer and T2D. But the TGF– *β* family would be involved in all the nine diseases. Besides the larger gene families such as the aforementioned three gene families, the proposed method also works on small gene family such as the IL–17 gene family which contains only six members. The relationship between gene expression and DNA methylation (promoter region or gene body region) mainly follows a bivariate OU model. The genes exhibiting significant dysregulation in promoter methylation and gene expressions are different with respect to the gene body methylation. In the TNF receptor family, most of the genes show significant alterations in promoter region than the gene body region, which is opposite in the TGF– *β* family. For the chemokine receptor family, the diseases Allergy, Asthma, CFS and Colitis involve both the gene body methylation and the promoter methylation of the family, but the diseases RA, SLE and T2D show differences in promoter methylation.

From biomedical literatures, we observe that the impact of methylation levels on diseases seems to be of the same magnitude as that of gene expression levels. Based on the identified disease associated genes for each gene family, the functional associations among the diseases based on the gene families are constructed, revealing the functional consistency and difference between diseases in terms of a signalling gene family. The members of the gene families exhibit different involvement in the inflammatory diseases. For example, viewing from the diseases gene network constructed based on the identified disease associated genes, the connectivities of the genes are different. For example, GDF15 is involved in seven diseases, while TGFB3, INHBC,A, TNFRSF1B and IL17E are associated with four diseases. Most of the other genes have two or three links to the diseases. We obtain multiple confirmatory results and a number of novel gene–disease associations that require experimental verification.

## Additional files

Additional file 1 Gene expression datasets of inflammatory diseases in Step 1. The table in the pdf file shows the public datasets of gene expression of the inflammatory diseases used in Step 1 of the analysis. (PDF 30 kb)

Additional file 2 DNA methylation and gene expression datasets of inflammation related diseases in Step 2. The table in the pdf file shows the public datasets of DNA methylation and gene expression of the inflammation related diseases used in Step 2 of the analysis. (PDF 42 kb)

Additional file 3 Significant genes for the inflammatory diseases analysed in Step 1. Tables in the Excel file show genes with significant *p*-values for different inflammatory diseases. Genes are sorted alphabetically in order to identify the gene families. (XLS 58 kb)

## Abbreviations

BM: Brownian motion
Colitis: Ulcerative Colitis
CFS: Chronic fatigue syndrome
Crohn: Crohns’Disease
DAG: Directed acyclic graph
DEGs: Dysregulated expressed genes
DMGs: Dysregulated methylated genes
eGWAS: Gene expression based genome–wide association study
GEO: Gene Expression Omnibus
GO: Gene ontology
HSCs: Hematopoietic stem cells
IBD: Inflammatory bowel disease
IC: Information content
IL–17: Interleukin 17
MICA: Most informative common ancestor
miRNA: microRNA
OU: Ornstein–Uhlenbeck
OUOU: Bivariate OU model
RA: Rheumatoid arthritis
T2D: Type 2 diabetes
TGF– *β*: Transforming growth factor beta
TNF: Tumor necrosis factor
SLE: Systemic lupus erythematosus
