# Supplementary material for: Multi–omic analysis of signalling factors in inflammatory comorbidities
Source: BMC Bioinformatics. 2018 Nov 30;19(Suppl 15):439. doi: 10.1186/s12859-018-2413-x (PMC6266935; doi:10.1186/s12859-018-2413-x)
Supplement: Supplementary file 1 — Gene expression datasets of inflammatory diseases in Step 1. The table in the pdf file shows the public datasets of gene expression of the inflammatory diseases used in Step 1 of the analysis. (PDF 30 kb) [file 12859_2018_2413_MOESM1_ESM.pdf]

Additional File 1

Gene expression datasets of inflammatory diseases used in Step 1

Disease	Expression Data Source	Cell Type
Osteoporosis	GSE7158	Osteoclast
HIV infection	GSE18464	Osteoclast
HIV infection with HAART therapy	GSE5220	Osteoclast
Osteoporosis	GSE35958	Osteoblast
HIV	GSE6740	CD4+ T cells
Rheumatoid arthritis	GSE1919	Synovial tissue
Osteoarthritis	GSE1919	Synovial tissue
Paget's disease	GSE30806, GSE9006	Quadriceps muscle, PBMC
Measles	GSE29106, GSE29107, GSE5808	Bone marrow, PBMC
Type 1 Diabetes	GSE9006	PBMC
Type 2 Diabetes	GSE9006	PBMC
Renal disorder	GSE29060	HT-29 cell line
Osteosarcoma	GSE21257	Bone
Multiple myeloma	GSE7390, GSE8977, GSE33450, GSE14548	Breast tumor, Mesenchymal stem cell, Epithelium
Breast cancer	GSE7390, GSE8977, GSE33450, GSE14548	Breast tumor, Mesenchymal stem cell, Epithelium
Periodontitis	GSE9006	PBMC
