# Supplementary material for: Multi–omic analysis of signalling factors in inflammatory comorbidities
Source: BMC Bioinformatics. 2018 Nov 30;19(Suppl 15):439. doi: 10.1186/s12859-018-2413-x (PMC6266935; doi:10.1186/s12859-018-2413-x)
Supplement: Supplementary file 2 — DNA methylation and gene expression datasets of inflammation related diseases in Step 2. The table in the pdf file shows the public datasets of DNA methylation and gene expression of the inflammation related diseases used in Step 2 of the analysis. (PDF 42 kb) [file 12859_2018_2413_MOESM2_ESM.pdf]

Additional File 2

DNA methylation and gene expression datasets of inflammation related diseases used in Step 2

Disease	Abbreviation	Methylation Data Source	Expression Data Source	Cell Type
Allergy	Allergy	GSE50387	GSE50387	CD4+ T cell
Asthma	Asthma	GSE52074	GSE18965	Airway epithelial cell
Ulcerative Colitis	Colitis	GSE32149	GSE3365	PBMC
Crohns' Disease	Crohn	GSE32149	GSE3365	PBMC
Rheumatoid Arthritis	RA	GSE42861	GSE15573	PBMC
Chronic Fatigue Syndrome	CFS	GSE59489	GSE14577	PBMC
Systemic Lupus Erythematosus	SLE	GSE59250	GSE46907	CD14+
Colon Cancer	ColonCancer	TCGA (Batch 76)	TCGA (Batch 28 and 76)	Colon
Type 2 Diabetes	T2D	Dayeh et al. [1]	GSE41762	Pancreatic islets

References

[1] Tasnim Dayeh, Petr Volkov, Sofia Salö, Elin Hall, Emma Nilsson, Anders H Olsson, Clare L Kirkpatrick, Claes B Wollheim, Lena Eliasson, Tina Rönn, et al. Genome-wide dna methylation analysis of human pancreatic islets from type 2 diabetic and non-diabetic donors identifies candidate genes that influence insulin secretion. *PLoS Genet*, 10(3):e1004160, 2014.
